# Liraglutide reduces plasma dihydroceramide levels in patients with type 2 diabetes

**DOI:** 10.1186/s12933-023-01845-0

**Published:** 2023-05-04

**Authors:** Damien Denimal, Victoria Bergas, Jean-Paul Pais-de-Barros, Isabelle Simoneau, Laurent Demizieux, Patricia Passilly-Degrace, Benjamin Bouillet, Jean-Michel Petit, Alexia Rouland, Amandine Bataille, Laurence Duvillard, Bruno Vergès

**Affiliations:** 1 University of Burgundy, INSERM LNC UMR1231, 21000 Dijon, France; 2 Department of Biochemistry, CHU Dijon Bourgogne, 21079 Dijon, France; 3 Lipidomic Analytical Platform, University of Burgundy, 21000 Dijon, France; 4 Department of Endocrinology and Diabetology, CHU Dijon Bourgogne, 21000 Dijon, France

**Keywords:** Type 2 diabetes, Liraglutide, Dihydroceramides, Ceramides

## Abstract

**Background:**

Emerging evidence supports that dihydroceramides (DhCer) and ceramides (Cer) contribute to the pathophysiology of insulin resistance and liver steatosis, and that their circulating concentrations are independently associated with cardiovascular outcomes. Circulating DhCer levels are increased in patients with type 2 diabetes (T2D). On the other hand, the GLP-1 receptor agonist liraglutide reduces major adverse cardiac events, insulin resistance and liver steatosis in T2D patients. The main purpose of the present study was therefore to investigate whether liraglutide decreases circulating levels of DhCer and Cer in T2D patients, which could be a mechanism involved in its cardiometabolic benefits. The secondary purpose was to assess the relationship between liraglutide-induced changes in DhCer/Cer levels and insulin resistance and liver steatosis.

**Methods:**

Plasma concentrations of 11 DhCer and 15 Cer species were measured by a highly-sensitive mass spectrometry system in 35 controls and 86 T2D patients before and after 6 months of liraglutide (1.2 mg/day). Insulin resistance was estimated by the triglyceride-glucose (TyG) index. Liver fat content (LFC) was assessed in 53 patients by proton magnetic resonance spectroscopy.

**Results:**

Plasma levels of total DhCer, 7 DhCer and 7 Cer species were increased in T2D patients compared to controls. Liraglutide decreased total DhCer by 15.1% (p = 0.005), affecting 16:0 (p = 0.037), 18:0 (p < 0.0001), 18:1 (p = 0.0005), 20:0 (p = 0.0003), 23:0 (p = 0.005) and 24:1 (p = 0.04) species. Total plasma Cer did not significantly change after liraglutide (p = 0.18), but 5 Cer species decreased significantly, *i.e.* 18:0 and 18:1 (both p < 0.0001), 19:0 and 24:1 (both p < 0.01) and 26:1 (p = 0.04). In multivariate analysis, the reduction in DhCer after liraglutide was independently associated with the reduction in LFC (p = 0.0005) and in TyG index (p = 0.05).

**Conclusions:**

Liraglutide reduces plasma levels of numerous DhCer and Cer species in T2D patients, which may contribute to the cardiovascular benefit observed in the LEADER trial. The independent association between the decrease in plasma DhCer level with the reduction in LFC and TyG index adds new insights regarding the relationship between DhCer, liver steatosis and insulin resistance.

*Trial registration* ClinicalTrials.gov identifier: NCT02721888.

**Supplementary Information:**

The online version contains supplementary material available at 10.1186/s12933-023-01845-0.

## Background

Dihydroceramides (DhCer) and ceramides (Cer) are bioactive sphingolipids that are implicated in several processes related to cardiometabolic diseases. Growing evidence suggests that they play a causative role in the pathophysiology of type 2 diabetes (T2D) and cardiovascular diseases [1]. One of the best-known negative effects of Cer is their ability to promote insulin resistance, and plasma Cer concentrations are positively associated with the degree of insulin resistance in humans [2–4]. In addition, recent studies reveal that circulating Cer levels are also strongly associated with cardiovascular outcomes, independently of traditional risk factors [5, 6].

DhCer have long been regarded only as inactive precursors of Cer. However, recent studies suggest that DhCer are directly involved in many processes relative to T2D and atherosclerosis, like insulin resistance [2]. Interestingly, plasma DhCer concentrations are increased in patients with coronary artery disease [7], and are linked to cardiovascular risk [8]. Moreover, circulating DhCer are inversely associated with the degree of insulin sensitivity [2, 9, 10], and are long-term strong predictors of T2D onset [11].

Emerging evidence indicates that Cer and DhCer also play a significant role in metabolic-associated fatty liver disease (MAFLD), which is highly prevalent during T2D. Cer are key mediators of several processes involved in MAFLD, such as hepatic insulin resistance, endoplasmic reticulum stress and mitochondrial dysfunction [12]. For instance, the inhibition of Cer synthesis in rats was found to protect against lipid accumulation in the liver [13]. Clinical studies reveal that circulating DhCer and Cer levels are increased in patients with MAFLD [10, 14], and that they mirror the hepatic content of DhCer and Cer [14]. In addition, it was recently reported that circulating DhCer concentrations are associated with the severity of liver steatosis in T2D patients whereas plasma Cer levels are not, suggesting that DhCer has a specific role [15].

Liraglutide, a glucagon-like peptide-1 receptor agonist (GLP-1 RA), significantly reduces the incidence of major adverse cardiovascular events (MACE) in the LEADER trial, and has beneficial effects on insulin resistance, liver steatosis and dyslipidemia in T2D patients [16–19]. Because DhCer and Cer play a role in the pathophysiology of cardiometabolic diseases and are associated with cardiovascular outcomes, the main purpose of the present study was to investigate whether liraglutide reduces the circulating levels of these lipids in T2D patients. Such a reduction may contribute to its cardiovascular and metabolic benefits. In addition, since DhCer and Cer play a role in the pathogenesis of MAFLD and insulin resistance, the secondary purpose of the study was to evaluate the relationship between the liraglutide-induced changes in circulating DhCer/Cer and liver fat content (LFC) and insulin resistance.

## Methods

### Subjects

This prospective single-center study was an ancillary study of the LIRA-NAFLD/LIP trial (ClinicalTrials.gov identifier: NCT02721888). We included 86 T2D patients treated with metformin and/or sulfonylurea (or glinides) and/or insulin, and for whom liraglutide was indicated because of poorly controlled diabetes (glycated hemoglobin A_1c_ [HbA_1c_] > 7%). Exclusion criteria were severe liver impairment, estimated glomerular filtration rate (eGFR) < 30 mL/min/1.73m^2^, pregnancy, alcohol abuse, treatment with dipeptidyl peptidase-4 inhibitors during the three previous months, or previous treatment with thiazolidinediones or any GLP-1 RA.

The results obtained in patients treated with liraglutide were compared to samples obtained from 35 healthy control subjects who had normal fasting glycemia (< 6.10 mmoL/L), normal triglyceridemia (< 1.7 mmoL/L), eGFR > 60 mL/min/1.73m^2^, and serum HDL-cholesterol (HDL-C) > 1.30 mmol/L for females and > 1.03 mmol/L for males. Exclusion criteria for control subjects were thyroid or kidney diseases, and the use of antidiabetic agents or medications known to affect lipid metabolism. This study was approved by our regional ethics committee, and written informed consent was obtained from all participants before study inclusion.

### Study design and intervention

At baseline, T2D patients had a physical examination, and blood samples were collected in tubes containing in particular EDTA as preservative for lipidomic analyses. The plasma/serum was immediately separated by centrifugation, and analyzed for routine parameters or frozen < − 70 °C awaiting lipidomic analyses. LFC was assessed in a subgroup of 53 T2D patients using a 3.0-Tesla magnetic resonance system (Magnetom Trio Tim; Siemens, Erlangen, Germany).

Treatment with liraglutide (Victoza™; Novo Nordisk A/S, Kalundborg, Denmark) was started the following day at a dose of 0.6 mg/day, which was up-titrated to 1.2 mg/day after one week until the end of the study. After six months of treatment, patients underwent the same series of examinations.

### Routine laboratory analyses

HbA_1c_ was measured by high performance liquid chromatography (Variant II; Biorad, Richmond, CA). Routine biochemical parameters were determined on a Dimension Vista platform (Siemens, Saint-Denis, France). LDL-cholesterol (LDL-C) was estimated by the Friedewald's equation. eGFR was calculated according to the 2021-CKD-EPI equation. The triglyceride-glucose (TyG) index, determined as ln(fasting triglycerides [mg/dL] × fasting glucose [mg/dL]/2), was chosen as the marker of insulin resistance since it performs better than HOMA-IR [20, 21].

### Plasma DhCer and Cer measurements

#### Lipid extraction

DhCer/Cer were extracted as previously described [22]. Briefly, 100 µL plasma (+ 100 µL PBS) were mixed with 10 µL of an internal standard mix (15 and 20 ng of 12:0 DhCer and Cer, respectively), then extracted with 750 µL of 1:2 chloroform/methanol for 10 min. Chloroform (250 µL) was then added and extraction continued for 10 min more. Water (250 µL) was then added and extraction continued for 10 min more. After centrifugation (9400 g, 5 min), the organic phase was collected. The aqueous phase was acidified with 8 µL of 3 mol/L of hydrochloric acid, and further extracted with 600 µL of chloroform for 10 min. After centrifugation (9400 g, 5 min), the two organic phases were combined, and washed with 800 µL of the upper phase from a chloroform/methanol/water (96.7:93.3:90) mixture. After centrifugation (9400 g, 5 min), the organic phase was evaporated under vacuum. Extracts were finally dissolved with 100 µL of 60:30:4.5 chloroform/methanol/water.

#### Liquid chromatography—tandem mass spectrometry (LC–MS/MS)

One microliter of extract was injected on a Vanquish Flex LC system coupled with a TSQ Altis MS/MS system (ThermoFisher Scientific). Separation was achieved on a Poroshell C8 column 2.1 × 3x100 mm, 2.7-µm (Agilent Technologies) using the following gradient conditions (0.3 mL/min): 1 min at 80% mobile phase B, 80% to 100% B over 8 min, 3 min at 100% B, then 5 min at 80% B. Mobile phases A and B consisted of water and methanol, respectively, both containing 1 mmol/L ammonium formate and 0.2% (v/v) formic acid. Individual DhCer and Cer species were identified using multiple-reaction monitoring (Additional file 1: Table S1). Calibration lines were obtained using external standards. Total lipids of each class (DhCer and Cer) were calculated by summing the individual lipid species.

### Statistical analysis

For each parameter, the modification from baseline after liraglutide treatment in T2D patients, named “delta”, was calculated as final value minus initial value. Statistics and graphs were performed using GraphPad Prism (version 9.1.1). Comparisons of proportions were performed using the chi-square test with Yates’ correction. Normality of continuous variables was assessed using the D'Agostino-Pearson test. Plasma DhCer and Cer levels and the delta after liraglutide treatment did not follow a Gaussian distribution, and this was not due to outliers (checked with the ROUT method). Data are therefore reported as medians [1^st^-3^rd^ quartiles]. The study required a sample size of 73 patients to achieve a power of 80% and a level of significance of 5%, for detecting a mean of the changes in DhCer levels of 50 nmol/L after treatment, assuming the standard deviation of the differences to be 150 nmol/L. We used nonparametric tests, which do not assume a Gaussian distribution. Thus, quantitative results between T2D patients and control subjects were compared using the Mann–Whitney U test. The paired Wilcoxon test was used for comparisons between baseline and after six months of liraglutide. For correlation analysis, univariate Spearman correlation coefficients (rho) were determined for continuous variables, and point biserial correlation was used to assess the correlations between continuous variables and categorical variables. Multivariate analyses were performed by multivariate linear regression. A Benjamini–Hochberg procedure was applied for controlling false positives in multiple testing, using a false discovery rate of 5%. A two-tailed probability level of 0.05 was considered statistically significant.

## Results

### Participant characteristics at baseline

Baseline characteristics of the 86 T2D patients and 35 control subjects are shown in Table 1. Patients had typical features of T2D, such as increased body mass index (BMI) (> 25 and > 30 kg/m^2^ for 98.8 and 79.1% of patients, respectively), elevated plasma triglyceride levels (≥ 1.70 mmol/L for 64.0% of patients), and low HDL-C concentrations (≤ 1.30 mmol/L for 64.9% of females and ≤ 1.03 mmol/L for 69.4% of males). Not surprisingly, the insulin resistance index TyG was higher in T2D patients than in controls (p < 0.0001).

**Table 1:** Clinical and biological characteristics

Characteristic	Controls	T2D patients—Liraglutide	
		At baseline (M0)	After 6 months of treatment (M6)
No. subjects	35	86	
Clinical characteristics			
Age (y)	44.0 [34.0–53.0]	58.7 [48.7–65.3]^****^	59.2 [49.2–65.8]^####^
Gender, F/M (n)	26 / 9	37 / 49^**^	
Diabetes duration (y)	NA	7.0 [2.0–17.0]	
Tabacco (present or past), n (%)	NP	45 (52%)	
Retinopathy, n (%)	NA	11 (13%)	
Nephropathy, n (%)	NA	15 (18%)	
BMI (kg/m^2^)	22.7 [20.5–24.6]	35.1 [31.1–40.8]^****^	34.1 [30.0–38.8]^####‡‡‡‡^
Metformin, n (%)	0	79 (92%)	
Sulfonylurea, n (%)	0	50 (58%)	
Insulin, n (%)	0	25 (29%)	
Statins, n (%)	0	33 (38%)	
Ezetimibe, n (%)	0	7 (8%)	
Fibrates, n (%)	0	8 (9%)	
Blood and hepatic markers			
HbA_1c_ (%)	NP	9.60 [8.35–11.05]	7.40 [6.35–8.25]^####^
Glucose (mmol/L)	4.83 [4.52–5.22]	9.33 [7.09–11.5]^****^	7.59 [5.99–9.14]^####‡‡‡‡^
eGFR (mL/min/1.73m^2^)	104 [91–116]	100 [87–107]	99 [88–108]
Triglycerides (mmol/L)	0.85 [0.62–1.05]	1.85 [1.51–2.72]^****^	1.69 [1.24–2.58]^##‡‡‡‡^
Total cholesterol (mmol/L)	5.08 [4.34–5.98]	4.59 [3.70–5.23]^**^	4.53 [3.77–5.23]^‡‡^
LDL-cholesterol (mmol/L)	3.08 [2.27–3.54]	2.45 [1.87–3.08]^**^	2.56 [1.81–3.05]^‡‡^
HDL-cholesterol (mmol/L)	1.70 [1.39–1.93]	1.01 [0.81–1.24]^****^	1.09 [0.93–1.38]^###‡‡‡‡^
TyG index	4.38 [4.26–4.50]	5.10 [4.89–5.32]^****^	4.91 [4.77–5.21]^####‡‡‡‡^
Liver fat content (%) ^1^	NP	17.3 [8.1–27.6]	10.5 [5.1–17.8]^####^

Data are medians [1st-3rd quartiles], otherwise indicated. eGFR, estimated glomerular filtration rate; BMI, body mass index; NA, not applicable; NP, not provided

^1^ LFC data are available in a subgroup of 53 T2D patients

Controls *vs.* T2D-Liraglutide M0: ^*^ p < 0.05, ^**^ p < 0.01, ^***^ p < 0.001, ^****^ p < 0.0001

Controls *vs.* T2D-Liraglutide M6: ^‡^ p < 0.05, ^‡‡^ p < 0.01, ^‡‡‡^ p < 0.001, ^‡‡‡‡^ p < 0.0001

T2D-Liraglutide M6 *vs.* M0: ^#^ p < 0.05, ^##^ p < 0.01, ^###^ p < 0.001, ^####^ p < 0.0001

### Plasma DhCer and Cer at baseline

**Fig. 1:**
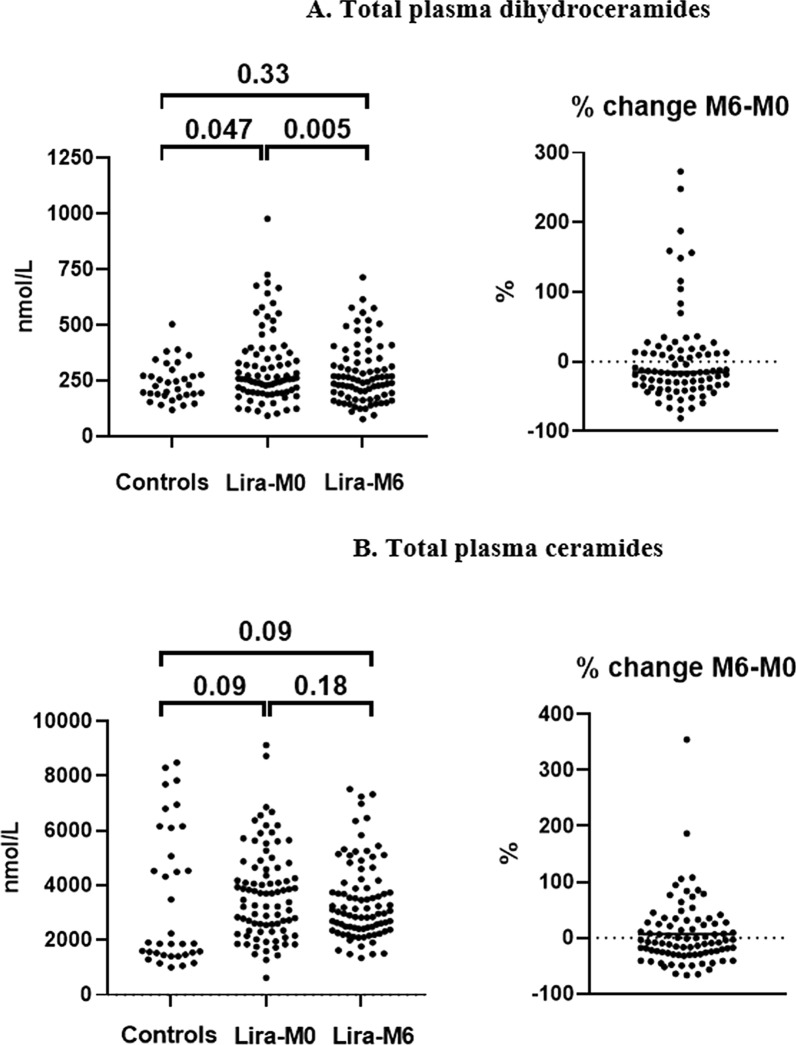
Plasma concentrations of total **A** dihydroceramides and **B** ceramides in control subjects and in patients with T2D at baseline (Lira-M0) and after 6 months of liraglutide (Lira-M6). Values correspond to p-values. The right panels represent the differences in percentage. The horizontal lines in the right panels represent medians

The median of total DhCer concentration in plasma was 12.7% higher in T2D patients at baseline than in controls (p = 0.047) (Table 2 and Fig. 1A). More precisely, T2D patients had higher plasma concentrations than controls for 7 of the 11 DhCer species we measured, *i.e.* 14:0. DhCer (p = 0.0001), 16:0 DhCer (p = 0.016), 18:0 DhCer (p < 0.0001), 18:1 DhCer (p < 0.0001), 20:0 DhCer (p < 0.0001), 22:0 DhCer (p = 0.0003) and 24:1 DhCer (p = 0.041). On the contrary, the levels of 26:0 and 26:1 DhCer were decreased in T2D patients (p-values of 0.013 and 0.012, respectively). The plasma concentrations of 23:0 and 24:0 DhCer were not significantly different between T2D patients and controls (p-values of 0.64 and 0.46, respectively).

**Table 2:** Plasma concentrations of dihydroceramides (DhCer) and ceramides (Cer)

Characteristic	Controls	T2D patients—Liraglutide		
		Baseline (M0)	After 6 months of treatment (M6)	% change M6-M0 [95%CI]
No. subjects	35	86		
Total DhCer	232 [187–299]	262 [201–395]^*^	254 [191–349]^###^	− 15.1 [− 4.7 to − 23.6]
14:0 DhCer	0.33 [0.18–0.48]	0.58 [0.45–0.76]^***^	0.55 [0.44–0.70]^‡‡‡^	− 5.5 [− 17.6 to 11.1]
16:0 DhCer	2.95 [2.64–3.71]	4.21 [2.88–5.56]^*^	3.81 [2.92–4.92]^#‡^	− 11.0 [− 21.9 to 1.9]
18:0 DhCer	4.8 [3.3–7.1]	13.1 [10.2–24.5]^****^	10.4 [8.13–17.5]^####‡‡‡‡^	− 26.0 [− 37.0 to − 13.9]
18:1 DhCer	0.74 [0.44–1.10]	3.52 [2.33–5.58]^****^	3.06 [1.86–4.16]^###‡‡‡‡^	− 19.9 [− 31.6 to − 10.5]
20:0 DhCer	3.31 [2.05–4.05]	9.55 [6.14–13.0]^****^	7.37 [5.01–10.7]^###‡‡‡‡^	− 17.9 [− 26.3 to − 0.2]
22:0 DhCer	35 [27–50]	52 [38–79]^***^	48 [35–67]^‡‡^	− 16.2 [− 23.6 to 3.4]
23:0 DhCer	37 [29–57]	41 [28–67]	35 [24–55]^##^	− 13.4 [− 32.1 to − 3.3]
24:0 DhCer	95 [59–153]	82 [56–143]	81 [49–123]	− 12.2 [− 21.8 to 2.4]
24:1 DhCer	43 [38–58]	54 [38–94]^*^	52 [40–73]^#^	− 15.8 [− 22.1 to 1.4]
26:0 DhCer	1.78 [0.23–3.72]	0.48 [0.23–1.12]^*^	0.44 [0.25–1.01]^‡‡^	− 5.1 [− 21.3 to 4.9]
26:1 DhCer	2.07 [0.11–4.34]	0.19 [0.06–0.44]^*^	0.19 [0.06–0.63]^‡^	− 7.2 [− 43.6 to 11.1]
Total Cer	1882 [1472–6100]	3585 [2353–4650]	3052 [2411–4118]	− 7.5 [− 16.5 to 6.4]
14:0 Cer	8.47 [5.95–17.2]	10.8 [8.75–13.4]	11.5 [8.72–13.8]	− 1.1 [− 7.6 to 11.2]
16:0 Cer	23.9 [20.8–30.9]	23.6 [20.0–27.0]	23.1 [20.2–26.6]	− 2.0 [− 7.1 to 7.44]
17:0 Cer	2.32 [1.92–2.73]	2.66 [2.29–3.13]	2.57 [2.12–2.95]	− 5.8 [− 11.7 to 5.1]
18:0 Cer	1.01 [0.52–22.2]	34.7 [21.7–46.1]^****^	30.5 [16.9–38.8]^####‡‡‡‡^	− 17.4 [− 23.4 to − 8.8]
18:1 Cer	1.11 [0.86–1.25]	1.43 [1.18–1.68]^***^	1.26 [0.99–1.42]^####^	− 16.0 [− 21.3 to − 7.2]
19:0 Cer	0.20 [0.11–2.02]	2.15 [1.51–2.62]^****^	1.94 [1.33–2.45]^##‡‡‡‡^	− 11.0 [− 19.9 to − 1.5]
20:0 Cer	8.7 [7.4–98]	83 [54–121]^**^	79 [47–110]^‡‡^	− 13.2 [− 18.7 to 8.1]
21:0 Cer	1.0 [0.6–15.7]	12.9 [6.5–17.8]^***^	12.6 [7.2–16.9]^‡‡‡^	− 6.0 [− 19.8 to 1.6]
22:0 Cer	269 [212–370]	292 [206–416]	296 [212–440]	− 1.3 [− 11.0 to 9.8]
23:0 Cer	221 [174–977]	651 [384–859]^*^	578 [404–895]	− 2.5 [− 13.2 to 12.9]
24:0 Cer	1145 [804–3052]	1793 [1155–2480]	1512 [1178–2259]	− 9.8 [− 19.5 to 7.2]
24:1 Cer	104 [79–569]	398 [294–578]^**^	355 [249–525]^##‡^	− 16.2 [− 20.0 to − 5.7]
25:0 Cer	106 [89–132]	74 [53–95]^****^	72 [48–99]^‡‡‡‡^	3.5 [− 12.7 to 18.3]
26:0 Cer	32.6 [19.3–56.9]	25.4 [18.1–32.6]^****^	21.5 [17.1–30.6]^‡‡^	− 7.1 [− 18.5 to 4.4]
26:1 Cer	12.9 [10.2–19.3]	16.3 [11.4–21.5]	14.4 [10.2–18.4]^#^	− 11.8 [− 17.0 to 3.3]

Data are expressed in nmol/L and correspond to medians [1^st^-3^rd^ quartiles], otherwise indicated

Controls vs. T2D-Liraglutide M0: ^*^ p < 0.05, ^**^ p < 0.01, ^***^ p < 0.001, ^****^ p < 0.0001

Controls vs. T2D-Liraglutide M6: ^‡^ p < 0.05, ^‡‡^ p < 0.01, ^‡‡‡^ p < 0.001, ^‡‡‡‡^ p < 0.0001

T2D-Liraglutide M6 vs. M0: ^#^ p < 0.05, ^##^ p < 0.01, ^###^ p < 0.001, ^####^ p < 0.0001

The total Cer level in plasma was increased by 90% in T2D patients at baseline compared with control subjects, but without reaching significance (difference of medians, p = 0.09) (Table 2 and Fig. 1B). However, the increase was statistically significant for 7 Cer species, i.e. 18:0 Cer (p < 0.0001), 18:1 Cer (p = 0.0004), 19:0 Cer (p < 0.0001), 20:0 Cer (p = 0.002), 21:0 Cer (p = 0.0001), 23:0 Cer (p = 0.02) and 24:1 Cer (p = 0.006). On the contrary, the plasma concentrations of 25:0 and 26:0 Cer were decreased in T2D patients (both p < 0.0001). The plasma levels of the remaining 6 Cer species were not statistically different between T2D patients and controls.

### Effects of six months of liraglutide treatment

The observed changes in the clinical and routine biological parameters after six months of treatment with liraglutide are shown in Table 1. BMI decreased significantly by 1.0 units [median of differences, 95% confidence interval (CI) 0.5–1.6 kg/m^2^, p < 0.0001], HbA_1c_ by 1.85 units (95%CI 1.40–2.50%, p < 0.0001), serum triglycerides by 0.19 mmol/L (95%CI 0.06–0.39 mmol/L, p = 0.002) and the TyG index by 2.6% (95%CI 1.2–4.0%, p < 0.0001). In the subgroup of the 53 T2D patients in whom a proton-spectroscopy of the liver was performed, the LFC significantly decreased from 17.3% (1^st^-3^rd^ quartiles, 8.1–27.6%) at baseline to 10.5% (5.1–17.8%) after liraglutide (p < 0.0001).

After treatment with liraglutide, total plasma concentration of DhCer significantly decreased by 15.1% (median of differences, 95%CI 4.7–23.6%), corresponding to a median difference of 48 nmol/L (95%CI 19–62 nmol/L, p = 0.005) (Table 2 and Fig. 1A). After treatment, the total DhCer concentration in patients was not different from that of control subjects (p = 0.33, Table 2 and Fig. 1A). As shown in Table 2, the decrease affected 6 DhCer species, reaching 11.0% for 16:0 DhCer (p = 0.037), 26.0% for 18:0 DhCer (95%CI 13.9–37.0%, p < 0.0001), 19.9% for 18:1 DhCer (95%CI 10.5–31.6%, p = 0.0005), 17.9% for 20:0 DhCer (95%CI 0.2–26.3%, p = 0.0003), 13.4% for 23:0 DhCer (95%CI 3.3–32.1%, p = 0.005) and 15.8% for 24:1 DhCer (95%CI 1.4–22.1%, p = 0.04). Plasma levels of 22:0, 24:0, 26:0 and 26:1 DhCer were not significantly different after liraglutide treatment compared to baseline.

Although the total plasma concentration of Cer was not statistically modified by liraglutide treatment (p = 0.18) (Table 2 and Fig. 1B), circulating concentrations were significantly lower after treatment for 5 Cer species. This decrease reached 17.4% for 18:0 Cer (95%CI 8.8–23.4%, p < 0.0001), 16.0% for 18:1 Cer (95%CI 7.2–21.3%, p < 0.0001), 11.0% for 19:0 Cer (95%CI 1.5–19.9%, p = 0.006), 16.2% for 24:1 Cer (95%CI 5.7–20.0%, p = 0.006) and 11.8% for 26:1 Cer (95%CI 3.3–17.0%, p = 0.04).

### Correlation analysis

**Fig. 2:**
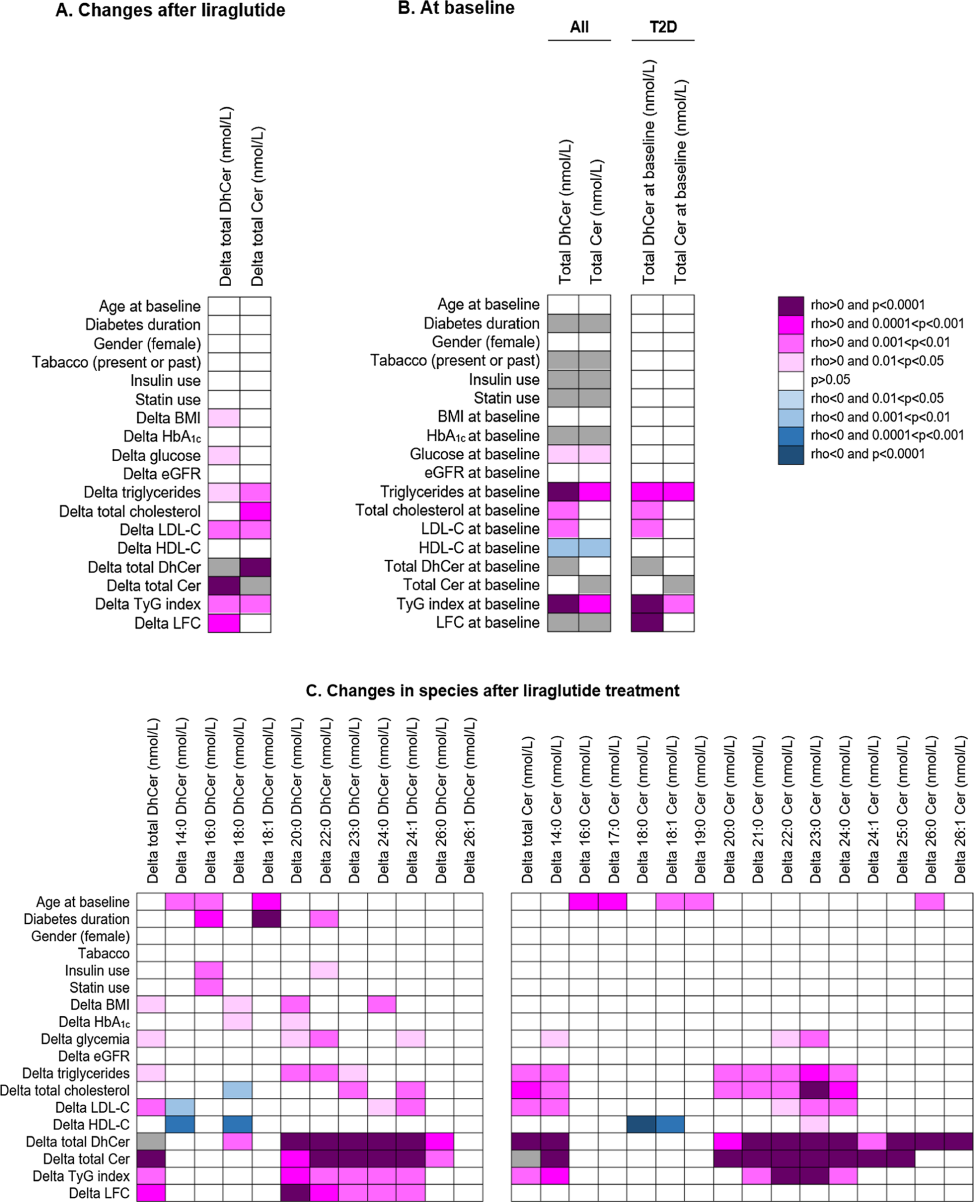
The heatmaps show univariate correlations **A** between changes in total plasma DhCer and Cer levels and modifications in clinical and biological parameters after six months of liraglutide treatment in T2D patients, **B** between total plasma DhCer and Cer levels and clinical and biological parameters at baseline, and **C** between changes in plasma levels of DhCer and Cer species and modifications in clinical and biological parameters after treatment in T2D patients. Only the correlations remaining significant after Benjamini–Hochberg correction to control the false discovery rate are reported in the heatmaps. Grey cells correspond to non-applicable calculations

Figure 2A shows the univariate correlations between changes in total plasma DhCer and Cer concentrations and changes in clinical and biological parameters after 6 months of treatment. The reduction in total DhCer level after liraglutide correlated positively with the decrease in total plasma Cer concentration (r = 0.619, p < 0.0001), in LFC (r = 0.493, p = 0.0002), in TyG index (r = 0.333, p = 0.002), in LDL-C (r = 0.280, p = 0.009) and in serum triglycerides (r = 0.265, p = 0.01). In multivariate analysis (Table 3), the decrease in total plasma DhCer concentration remained significantly associated with the reduction in LFC (β = 0.576, 95%CI 0.266–0.886, p = 0.0005), in total plasma Cer concentration (β = 0.489, 95%CI 0.192–0.786, p = 0.002), in serum triglycerides (β = 0.586, 95%CI 0.093–1.079, p = 0.021), in plasma glucose (β = 0.369, 95%CI 0.015–0.724, p = 0.04) and in TyG index (β = 0.126, 95%CI 0.020–0.232, p = 0.05). Interestingly, the baseline total plasma DhCer concentration in T2D subjects was strongly and positively associated with the baseline LFC (r = 0.468, p = 0.0002) and TyG index (r = 0.400, p = 0.0002) (Fig. 2B). Besides total DhCer, the significant decreases after liraglutide in the 20:0, 23:0 and 24:1 DhCer species were all positively associated with the reduction in both LFC and TyG index (Fig. 2C).

**Table 3:** Multivariate linear regression analysis in T2D patients

Variables	Standardized coefficients [95% confidence interval]	P-value
Delta total plasma DhCer		
Delta LFC	0.576 [0.266–0.886]	0.0005
Delta total plasma Cer	0.489 [0.192–0.786]	0.002
Delta serum triglycerides	0.586 [0.093–1.079]	0.021
Delta plasma glucose	0.369 [0.015–0.724]	0.04
Delta TyG index	0.126 [0.020–0.232]	0.05
Delta BMI	− 0.254 [− 0.570–0.063]	0.11
Delta serum LDL-C	0.064 [− 0.220–0.347]	0.65
Delta total serum cholesterol	− 0.034 [− 0.335–0.266]	0.82
Delta HbA_1c_	− 0.027 [− 0.352–0.299]	0.87
Delta TyG index		
Delta serum triglycerides	0.639 [0.488–0.791]	< 0.0001
Delta plasma glucose	0.431 [0.295–0.566]	< 0.0001
Delta LFC	0.228 [0.100–0.356]	0.0009
Delta HbA_1c_	0.171 [0.023–0.319]	0.02
Delta total plasma DhCer	0.149 [0.006–0.292]	0.04
Delta eGFR	0.020 [− 0.128–0.168]	0.78
Diabetes duration	− 0.008 [− 0.127–0.112]	0.90
Delta total plasma Cer	0.010 [− 0.165–0.185]	0.91
Delta serum LDL-C	0.007 [− 0.132–0.147]	0.91
Delta LFC		
Delta BMI	0.540 [0.316–0.765]	< 0.0001
Delta total plasma DhCer	0.442 [0.209–0.675]	0.0004
Delta TyG index	0.665 [0.143–1.188]	0.01
Delta total plasma Cer	− 0.206 [− 0.477–0.066]	0.13
Delta serum triglycerides	− 0.319 [− 0.757–0.120]	0.15
Delta plasma glucose	− 0.212 [− 0.520–0.095]	0.17
Delta HbA_1c_	0.044 [− 0.234–0.322]	0.75

All variables with a raw p-value < 0.10 in univariate regression analysis were included in the model

**Fig. 3:**
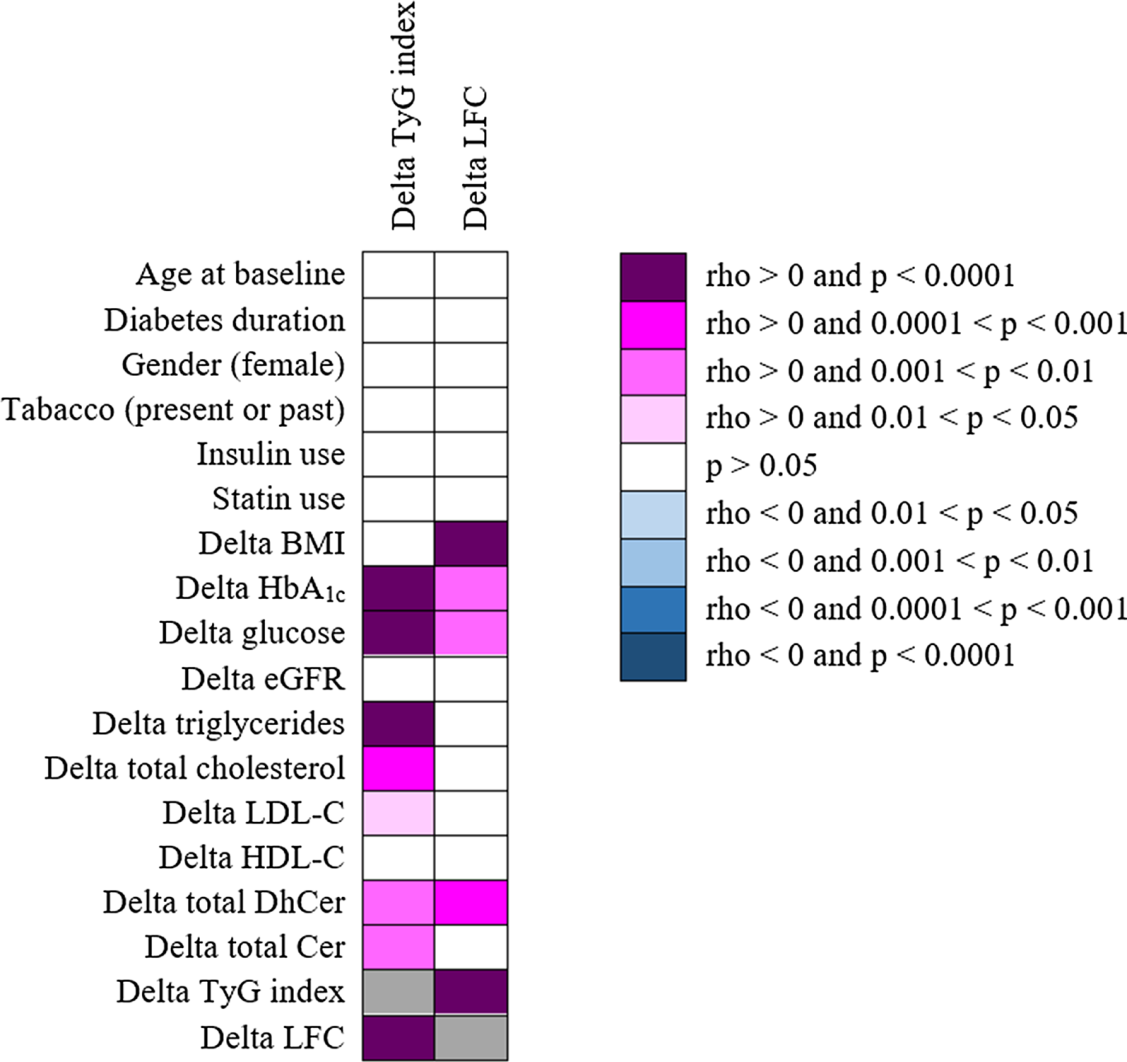
The heatmap show univariate correlations between reduction in TyG index and LFC after six months of liraglutide treatment in T2D patients and changes in clinical and biological parameters. Only the correlations remaining significant after Benjamini–Hochberg correction to control the false discovery rate are reported in the heatmap. Grey cells correspond to non-applicable calculations

In univariate regression analysis, the reduction in TyG index after liraglutide treatment correlated positively with the decrease in plasma glucose (r = 0.757, p < 0.0001), serum triglycerides (r = 0.718, p < 0.0001), LFC (r = 0.531, p < 0.0001), HbA_1c_ (r = 0.494, p < 0.0001), serum total cholesterol (r = 0.399, p = 0.0002), total plasma Cer (r = 0.346, p = 0.001) and total plasma DhCer (r = 0.333, p = 0.002) (Fig. 3). Interestingly, in multivariate analysis the reduction in TyG index remained significantly associated with the decrease in total plasma DhCer (β = 0.149, 95%CI 0.006–0.292, p = 0.04) (Table 3).

In univariate regression analysis, the reduction in LFC after liraglutide treatment correlated positively with the decrease in TyG index (r = 0.531, p < 0.0001), BMI (r = 0.524, p < 0.0001), plasma DhCer (r = 0.493, p = 0.0002), plasma glucose (r = 0.431, p = 0.001) and HbA_1c_ (r = 0.413, p = 0.002) (Fig. 3). Interestingly, the reduction in LFC was strongly associated with the decrease in total plasma DhCer (β = 0.442, 95%CI 0.209–0.675, p = 0.0004) in multivariate analysis, independently of changes in BMI, HbA_1c_, serum triglycerides and total plasma Cer level (Table 3).

## Discussion

In the present study, we report for the first time that liraglutide treatment significantly reduces the plasma concentrations of numerous DhCer species in patients with T2D. Firstly, we found that T2D patients had an increased total plasma DhCer at baseline compared with controls. A similar result has already been reported, but with a smaller number of either T2DM patients or control subjects and with a lower number of quantified DhCer species [2, 23]. Thus, Zarini et al*.* found increased plasma levels of 18:0, 20:0, 22:0 and 24:1 DhCer in T2D patients [2]. Our results are also consistent with those of Szpigel et al*.*, except for the 24:1 DhCer for which they found a decrease in T2D [23]. However, the 24:1 DhCer species was found to be increased in T2D adolescents [24]. We observed no difference in total plasma Cer level between T2D patients and controls. Previous studies reported either a similar observation [2, 23], or a slight increase in total plasma Cer in T2DM patients [3]. Increased plasma levels of 18:0, 20:0 and 24:1 Cer we observed have already been reported in T2D patients [3, 25].

After six months of liraglutide treatment, the T2D patients in our cohort exhibited typical features of this treatment, including lower HbA_1c_ and weight loss. While there was no change in total plasma Cer levels, we did observe a significant decrease in the plasma levels of 5 Cer species after liraglutide. Using the short-acting GLP-1 RA exenatide in 35 T2D patients, Zhang et al*.* recently found no reduction in plasma levels of Cer species, but exenatide is less potent for metabolic control than liraglutide [26], and the treatment duration was only 3 months compared to the 6 months of liraglutide in our study [25]. Using a metabolomic approach in 33 T2D patients, Jendle et al*.* recently reported that 18 weeks of a treatment with liraglutide induced no significant change in total plasma Cer levels, like in our study, but unfortunately they did not provide data on individual Cer species [27]. Zobel et al*.* found a reduction in 19:0 and 20:0 Cer species in 51 T2D patients after 26 weeks of treatment with liraglutide, but without reporting the levels of total Cer [28]. The decrease in plasma 24:1 Cer we observed is of particular interest, since the circulating concentration of this species predicts incident MACE independently of traditional cardiovascular risk factors in general [29], high cardiovascular risk [6], and T2D population [30]. The decrease in 18:0 Cer we observed after liraglutide is also noteworthy since an increase in one standard deviation of plasma 18:0 Cer concentration was associated with a 21% increase in MACE in the FinRisk study, independently of traditional cardiovascular risk factors [29].

Regarding DhCer, we reported for the first time that the plasma levels of total, 16:0, 18:1, 20:0, 23:0 and 24:1 DhCer significantly decreased in T2D patients after liraglutide treatment, which is particularly interesting since their plasma concentrations were reported to be associated with incident cardiovascular diseases, even after adjustment for traditional cardiovascular risk factors [7, 8]. For instance, an increase of one standard deviation in total plasma DhCer level was associated with a 146% increase in the risk of coronary artery disease [7]. To the best of our knowledge, only one study reported that liraglutide decreases circulating levels of 24:0 DhCer, but they did not provide data on other DhCer species [28]. Overall, the liraglutide-induced reduction in numerous DhCer and Cer species could be one of the mechanisms contributing to the beneficial cardiovascular effects of this treatment.

The mechanisms underlying the decrease in numerous DhCer/Cer species are intriguing. The fact that reductions in DhCer and Cer levels after liraglutide treatment were strongly and positively associated suggests that liraglutide acts on a step in the metabolism that is in common with both DhCer and Cer. DhCer are produced by the canonical de novo Cer synthesis pathway, and it may be hypothesized that liraglutide downregulates this pathway. In agreement with this hypothesis, Somm et al*.* recently found that liraglutide administered in mice reduces the liver gene expression of the serine palmitoyltransferase (SPT)-2, involved in the first step of de novo DhCer/Cer synthesis [31]. The expression of SPT was also downregulated after treatment of cardiac cells with exenatide [32]. Liraglutide-treated mice exhibited a decreased liver gene expression of Cer synthase-4, which preferentially adds C18 to C20 fatty acids to the sphingoid basis, in agreement with the liraglutide-induced reduction in plasma C18, C19, C20 DhCer and Cer that are reported in our study.

The significant decrease observed in the insulin resistance index TyG after treatment is consistent with previous studies showing that liraglutide improves insulin sensitivity in T2D patients [33]. Interestingly, we report for the first time that the decrease in total plasma DhCer level after liraglutide was positively and independently associated with the reduction in the TyG index in our cohort. In addition, total plasma DhCer concentration at baseline was positively and strongly associated with the TyG index. Similarly, recent studies reported that total plasma DhCer in humans was inversely associated with insulin sensitivity, measured with a hyperinsulinemic euglycemic clamp [2, 10]. In addition to total DhCer, the significant decrease in 20:0 and 24:1 DhCer after liraglutide treatment correlated with the reduction in the TyG index in our study, and it has been shown that the circulating levels of these two DhCer were negatively associated with insulin sensitivity in a recent study [2]. The relationship between DhCer and insulin resistance may be causative, since a recent study reported that the delivery of DhCer carried in liposomes into skeletal muscle cells counteracts the stimulating effect of insulin on glycogen synthesis, without affecting cellular Cer levels [2]. Regarding Cer, their role in the pathogenesis of insulin resistance is better established, and it is known that they are able to antagonize the insulin-dependent glucose uptake. The decrease in plasma 18:0 Cer we observed after liraglutide is notable because the plasma level of this species is inversely associated with insulin sensitivity in humans [2]. Taken together, these data suggest that the reduction in circulating levels of numerous Cer and DhCer species might contribute to the improvement of insulin sensitivity in liraglutide-treated T2D patients.

Liver steatosis was significantly reduced after liraglutide treatment in our study, which is concordant with previous reports [17]. Interestingly, we report for the first time that the reduction in LFC positively and independently correlated with the decrease in total plasma DhCer in multivariate analysis, but not with the change in Cer. Moreover, plasma DhCer level at baseline was strongly associated with LFC, unlike Cer. Besides total DhCer, the significant decrease in 20:0 and 24:1 DhCer after liraglutide correlated with the reduction in LFC in our study. Accordingly, the circulating levels of the two species have been shown to be positively associated with histological liver steatosis in obese individuals [14] and with the non-invasive SteatoTest™ in T2D patients [15]. In another study including T2D patients, Carlier et al*.* also found that total plasma DhCer levels correlate with the intensity of liver steatosis assessed by SteatoTest™, whereas total plasma Cer levels do not [15]. Interestingly, the inhibition in human liver cells of the DhCer desaturase, which converts DhCer into Cer, upregulated triglyceride storage in lipid droplets in relation with an increased level of DhCer [34]. All of these findings suggest that DhCer have a specific role independent from Cer in the pathophysiology of liver steatosis in T2D.

The strengths of our study include the novelty of reporting the effect of liraglutide treatment in T2D patients on circulating DhCer, which are increasingly seen as important bioactive sphingolipids involved in many processes occurring in T2D and atherosclerosis. Using a highly sensitive LC–MS/MS, we quantified a panel of DhCer and Cer species that is more extensive than the panels usually reported in clinical studies. Another strength is the novelty of investigating the association between changes in plasma DhCer/Cer after liraglutide treatment and those in LFC and the TyG index, thus providing new insights into the relationship between these sphingolipids and liver steatosis and insulin resistance in T2D. A limitation of our study is the lack of histological measures of liver steatosis. However, we assessed the LFC with proton-spectroscopy, which is recognized as a gold-standard method. Another limitation is the lack of in vivo measurements of insulin sensitivity, such as with a euglycemic insulin clamp. However, we used TyG index as surrogate, which is a strong predictor of MACE [21], and a reliable marker of insulin resistance that performs better than HOMA-IR [20]. Lastly, the age and sex ratio were different between controls and patients. However, we assume that this has no impact on our results since the changes in total plasma DhCer/Cer levels after liraglutide did not correlate with age and gender, and no correlation occurred at baseline in T2D patients and all subjects.

## Conclusion

Liraglutide significantly reduces plasma levels of numerous DhCer and Cer species in patients with T2D, which is of particular interest due to the emerging role of these lipids in the pathophysiology of cardiometabolic diseases and their association with cardiovascular outcomes. Further investigations are needed to determine whether the measurement of Cer/DhCer levels is of additional interest in clinical practice, in T2D patients with MAFLD regarding cardiovascular and steatosis risks. In addition, these metrics could be relevant outcomes to consider during the development of new antidiabetic medications.

## Supplementary Information

Additional file 1: Table S1. Quantified dihydroceramide and ceramide species and conditions for mass spectrometry acquisition. CE, collision energy; IS, internal standard used for quantitation.

## Data Availability

The datasets generated during and/or analysed during the current study are available from the corresponding author on reasonable request.

## Abbreviations

BMI: Body mass index
Cer: Ceramide
DhCer: Dihydroceramide
eGFR: Estimated glomerular filtration rate
GLP-1 RA: Glucagon-like peptide-1 receptor agonist
LFC: Liver fat content
MACE: Major adverse cardiovascular event
MAFLD: Metabolic-associated fatty liver disease
SPT: Serine palmitoyl-transferase
T2D: Type 2 diabetes
TyG: Triglyceride-glucose
