# Supplementary material for: Liraglutide reduces plasma dihydroceramide levels in patients with type 2 diabetes
Source: Cardiovasc Diabetol. 2023 May 4;22:104. doi: 10.1186/s12933-023-01845-0 (PMC10158384; doi:10.1186/s12933-023-01845-0)
Supplement: Supplementary file 1 — Additional file 1: Table S1. Quantified dihydroceramide and ceramide species and conditions for mass spectrometry acquisition. CE, collision energy; IS, internal standard used for quantitation. [file 12933_2023_1845_MOESM1_ESM.docx]

**Additional files**

**Table S1.** Quantified dihydroceramide and ceramide species and conditions for mass spectrometry acquisition. CE, collision energy; IS, internal standard used for quantitation.

| **Molecular species** | **Mass transition (m/z)** | **CE**  **(eV)** | **Retention time**  **(min)** |
| --- | --- | --- | --- |
| **Dihydroceramides** | | | |
| d18:0/12:0 (IS) | 484.6⭢266.1 | 25 | 8.1 |
| d18:0/14:0 | 512.5⭢266.3 | 25 | 8.9 |
| d18:0/16:0 | 540.7⭢266.2 | 25 | 9.5 |
| d18:0/18:0 | 568.8⭢266.1 | 25 | 10.1 |
| d18:0/18:1 | 566.6⭢266.3 | 25 | 9.7 |
| d18:0/20:0 | 596.4⭢266.3 | 25 | 10.6 |
| d18:0/22:0 | 624.6⭢266.3 | 25 | 11.1 |
| d18:0/23:0 | 638.6⭢266.3 | 25 | 11.3 |
| d18:0/24:0 | 652.8⭢266.1 | 25 | 11.6 |
| d18:0/24:1 | 650.8⭢266.2 | 25 | 11.3 |
| d18:0/26:0 | 680.4⭢266.3 | 25 | 12.1 |
| d18:0/26:1 | 678.4⭢266.3 | 25 | 11.7 |
|  |  |  |  |
| **Ceramides** | | | |
| d18:1/12:0 (IS) | 464.3⭢264.1 | 25 | 7.9 |
| d18:1/14:0 | 492.6⭢264.1 | 25 | 8.6 |
| d18:1/16:0 | 520.6⭢264.1 | 25 | 9.3 |
| d18:1/17:0 | 534.6⭢264.1 | 25 | 9.6 |
| d18:1/18:0 | 548.6⭢264.1 | 25 | 9.9 |
| d18:1/18:1 | 546.5⭢264.3 | 25 | 9.5 |
| d18:1/19:0 | 562.5⭢264.3 | 25 | 10.1 |
| d18:1/20:0 | 576.8⭢264.2 | 25 | 10.4 |
| d18:1/21:0 | 590.6⭢264.3 | 25 | 10.7 |
| d18:1/22:0 | 604.7⭢264.2 | 25 | 10.9 |
| d18:1/23:0 | 618.6⭢264.3 | 25 | 11.2 |
| d18:1/24:0 | 632.7⭢264.1 | 25 | 11.4 |
| d18:1/24:1 | 630.8⭢264.1 | 25 | 11.1 |
| d18:1/25:0 | 646.7⭢264.3 | 25 | 11.7 |
| d18:1/26:0 | 660.7⭢264.3 | 25 | 11.9 |
| d18:1/26:1 | 658.7⭢264.3 | 25 | 11.5 |
